# RBM25 Mediates Abiotic Responses in Plants

**DOI:** 10.3389/fpls.2017.00292

**Published:** 2017-03-10

**Authors:** Chunhong Cheng, Zhijuan Wang, Bingjian Yuan, Xia Li

**Affiliations:** ^1^Center for Agricultural Resources Research, Institute of Genetics and Developmental Biology, Chinese Academy of SciencesShijiazhuang, China; ^2^University of Chinese Academy of SciencesBeijing, China; ^3^State Key Laboratory of Agricultural Microbiology, College of Plant Science and Technology, Huazhong Agricultural UniversityWuhan, China

**Keywords:** RBM25, splicing factor, *Arabidopsis*, drought, abiotic stresses

## Abstract

Alternative splicing (AS) of pre-mRNAs is one of the most important post-transcriptional regulations that enable a single gene to code for multiple proteins resulting in the biodiversity of proteins in eukaryotes. Recently, we have shown that an *Arabidopsis thaliana* RNA recognition motif-containing protein RBM25 is a novel splicing factor to modulate plant response to ABA during seed germination and post-germination through regulating *HAB1* pre-mRNA AS. Here, we show that *RBM25* is preferentially expressed in stomata and vascular tissues in *Arabidopsis* and is induced by ABA and abiotic stresses. Loss-of-function mutant is highly tolerant to drought and sensitive to salt stress. Bioinformatic analysis and expression assays reveal that *Arabidopsis RBM25* is induced by multiple abiotic stresses, suggesting a crucial role of *RBM25* in *Arabidopsis* responses to adverse environmental conditions. Furthermore, we provide a comprehensive characterization of the homologous genes of *Arabidopsis RBM25* based on the latest plant genome sequences and public microarray databases. Fourteen homologous genes are identified in different plant species which show similar structure in gene and protein. Notably, the promoter analysis reveals that *RBM25* homologs are likely controlled by the regulators involved in multiple plant growth and abiotic stresses, such as drought and unfavorable temperature. The comparative analysis of general and unique *cis* regulatory elements of the *RBM25* homologs highlights the conserved and unique molecular processes that modulate plant response to abiotic stresses through RBM25-mediated alternative splicing.

## Introduction

As plants are essentially sessile in nature, they have to face various environmental stresses during life time. Drought and high salinity are two of the most important factors that greatly affect plant growth, yield, and distribution. Global warming increases the frequency and severity of extreme weather that exacerbate the effects of abiotic stresses (i.e., drought, cold, and heat stresses) on plants. To deal with these challenges, plants have evolved numerous adaptive strategies at physiological, biochemical and molecular levels in order to survive better (Broun, 2004; Bohnert et al., 2006). Plant stress responses are regulated by numerous gene regulatory networks at multiple levels. In the past decades, the extensive efforts have been made to understand how plants respond to abiotic stresses at transcriptional level. However, how plants respond to abiotic stresses at post-transcriptional level is less clear.

Pre-mRNA splicing is a process that removes the introns and joins exons to produce mature mRNA. It is one of the important post-transcriptional regulation events that control gene expression and plant response to developmental and environmental stresses (Reddy, 2007). Alternative splicing (AS) produces transcript isoforms that encode proteins with different subcellular localization, stability and functions (Chung et al., 2010; Kriechbaumer et al., 2012; Remy et al., 2013). It has been shown that more than 61% genes in *Arabidopsis* undergo AS in normal conditions and the percentage increases greatly under stress conditions (Marquez et al., 2012). AS is carried out in a large complex called spliceosome, where ribonucleoprotein particles (snRNPs) and numerous other snRNP-associated proteins are assembled precisely and orderly (Makarova et al., 2001). A large number of splicing factors are involved in pre-mRNA splicing and are necessary for generating mature mRNAs. Recent research has discovered that some plant splicing factors play important roles in plant development and stress adaptation. The plant specific splicing factor SR45 functions in various developmental processes. The *sr45* mutant shows defects in flowering, leaf and root development (Ali et al., 2007). SUPPRESSOR OF *abi3-5* (SUA) affects seed maturation by regulating AS of *ABSCISIC ACID INSENSITIVE3* (*ABI3*) (Sugliani et al., 2010). Loss-of-function in STA1, a pre-mRNA splicing factor, caused defects in the splicing of the cold-induced *COR15A* (*COLD-REGULATED 15A*) and subsequent altered stress response of plants (Lee et al., 2006). Mutations in *SR34B* affect splicing of *Iron-regulated Transporter 1* (*IRT1*) pre-mRNAs under Cd stress to reduce the *IRT1* mRNA accumulation resulting in increased sensitivity of plants to Cd stress (Zhang et al., 2014). Furthermore, it has been shown that AS of some stress-responsive genes is crucial for their functions in plant stress response to abiotic stress. For example, rice *DEHYDRATION-RESPONSIVE ELEMENT BINDING PROTEIN2* (*OsDREB2B*), which is temperature- and drought-responsive, undergoes pre-mRNA AS that is required for its function in plant response to low temperature and osmotic stress (Matsukura et al., 2010). Thus, splicing factors and AS of pre-mRNAs play a fundamental role in plant development and plant stress adaptation.

Recently, we have shown that RBM25, an RNA-recognition motif (RRM) containing protein, acts as a key regulator of *HAB1* pre-mRNA AS in *Arabidopsis* (Wang et al., 2015). RBM25 can bind to the intron of *HAB1* to yield the opposite functional isoforms *HAB1.2* and *HAB1.1*, and the ratio of *HAB1.2/HAB1.1* regulates the switching off and on of the ABA signaling pathway that modulates seed germination and post-germinative growth in response to ABA treatment (Wang et al., 2015). However, many questions still remain unknown, such as whether RBM25 is involved in other biological progresses, how the *RBM25* gene(s) is likely regulated and whether the *RBM25* homologes genes/proteins share similar functions. Here, we show that *RBM25* is preferentially expressed in vascular tissues in *Arabidopsis* and is induced by ABA, salt, drought, and other abiotic stresses. Intriguingly, mutation in *RBM25* dramatically enhances plant tolerance to drought and salt stress. Moreover, we show that *Arabidopsis RBM25* and its homologous genes share the highly conserved gene structures and protein motifs, indicating that they are involved in similar molecular processes and biological functions in plant growth and response to abiotic stresses. Notably, our data reveal that RBM25 is likely to mediate plant response to more abiotic stresses and to be regulated by manifold regulators that are involved in plant responsiveness to multiple plant growth regulators. Our results provide important and novel insights into the regulation of the *Arabidopsis RBM25* and its homologous genes and the potential roles in regulating abiotic stress responses in plants.

## Materials and Methods

### Plant Materials and Growth Conditions

*Arabidopsis thaliana* ecotype Columbia-0 (Col-0), T-DNA insertion mutant *rbm25-1* and the complimented lines *RBM25-9, RBM25-10* were used in this study (Wang et al., 2015). Seeds were sterilized with 50% bleach, washed five times using sterile water, and then sowed on MS medium containing 1% sucrose and 0.8% agar. The plates were stratified in darkness at 4°C for 2 days and then transferred to culture room at 22°C under a 16 h of light/8 h of dark photoperiod. After 7 days, the seedlings were planted in soil.

### Gene Expression Analysis

For the expression assay of *HAB1.2/HAB1.1*, total RNA was extracted using Trizol reagent (Life Technologies, Carlsbad, CA, USA) from 3-week-old seedlings exposed in air for different time. Equal amounts of RNA samples were used for reverse transcription with RevertAid^TM^ First Strand cDNA Synthesis Kit (Invitrogen) according to the manufacturer’s instructions. *ACTIN2* was used as the internal control for quantitative RT-PCR (qRT-PCR). The relative expression levels of the target genes were calculated by the equation Y = 2^Ct^ (Ct is the difference in Ct between the target and control products, i.e., Ct = Ct*_HAB1.1_*-Ct*_ACTIN_*).

For the expression assay of *RBM25*, total RNA was extracted from 10-day-old Col-0 seedlings treated with different abiotic stresses. qRT-PCR was performed as described above, primers used are listed in Supplementary Table S1.

### Phenotypic Analysis Assay

For phenotypic analysis in the presence of NaCl and Mannitol, MS medium was prepared supplemented with 1% sucrose and different concentrations of NaCl and Mannitol. Seeds were surface-sterilized and sowed on the MS medium with NaCl or Mannitol (three plates per treatment). The plates were stratified at 4°C for 2 days and then transferred to chamber at 22°C. Seedlings with elongated radicles or with green cotyledons were counted at the indicated time points.

### GUS Histochemical Analysis

For the GUS histochemical analysis, a 3-kb DNA fragment upstream of *RBM25* ATG start codon was cloned into pCAMBIA1391 to get *P_RBM25_*::*GUS*, using primers listed in Supplementary Table S1. The *P_RBM25_*::*GUS* construct was introduced into *Agrobacterium tumefaciens* strain GV3101 and then transformed into Col-0. The GUS assay was performed as previously described (Zhao et al., 2011). The seeds or seedlings were harvested and treated with MS or MS containing 50 uM ABA for 3 h or treated with different abiotic stresses. The histochemical staining was conducted by incubating those materials in freshly prepared buffer containing 5-bromo-4-chloro-3-indolyl-b-D-glucuronic acid for specified time at 37°C in the dark followed by clearing with 75% ethanol. The GUS-staining image was taken by optical microscope.

### Drought Treatment and Stomatal Aperture Measurement

For the drought tolerance assay, 2-week-old seedlings were withheld from water for 21 days, and then survival rates were determined and the plants were re-watered. To analyze stomatal function, rosette leaves were incubated under light in the solution containing 50 mM KCl, 10 mM CaCl_2_, and 10 mM MES (pH 6.15) for at least 2 h to keep the stoma open totally. Then ABA was added to a final concentration of 10 uM. After ABA treatment for 3 h, stomatal apertures were measured as described previously (Ren et al., 2010).

### Phylogenetic Tree and Gene Structure Analysis

We obtained the sequences of the identified *Arabidopsis RBM25* and its homologous genes from a published database phytozome^1^, including genomic DNA sequences, coding sequences, and amino acid sequences. A phylogenetic tree was constructed based on the amino acid sequences of *Arabidopsis* RBM25 and its homologous proteins using MEGA6. The gene structures of *Arabidopsis RBM25* and its homologous genes were determined using the Gene Structure Display Server (GSDS) website^2^.

### Promoter Analysis

Sequences of a 3-kb fragments upstream of *Arabidopsis RBM25* and its homologous genes were downloaded from website. The regulatory *cis*-elements of *Arabidopsis RBM25* and its homologous genes were then analyzed using PlantCARE^3^.

### Statistical Analysis

All data were analyzed using SigmaPlot 10.0 (Systat Software, Inc., Chicago, IL, USA). The averages and standard deviations of all results were calculated, and Student’s *t*-test were performed to generate *P*-values. And the statically significant treatments were marked with (^∗∗^*P* < 0.01). ns, no significant (*P* > 0.05).

## Results

### *RBM25* Is Expressed Ubiquitously in All Tissues

**FIGURE 1:**
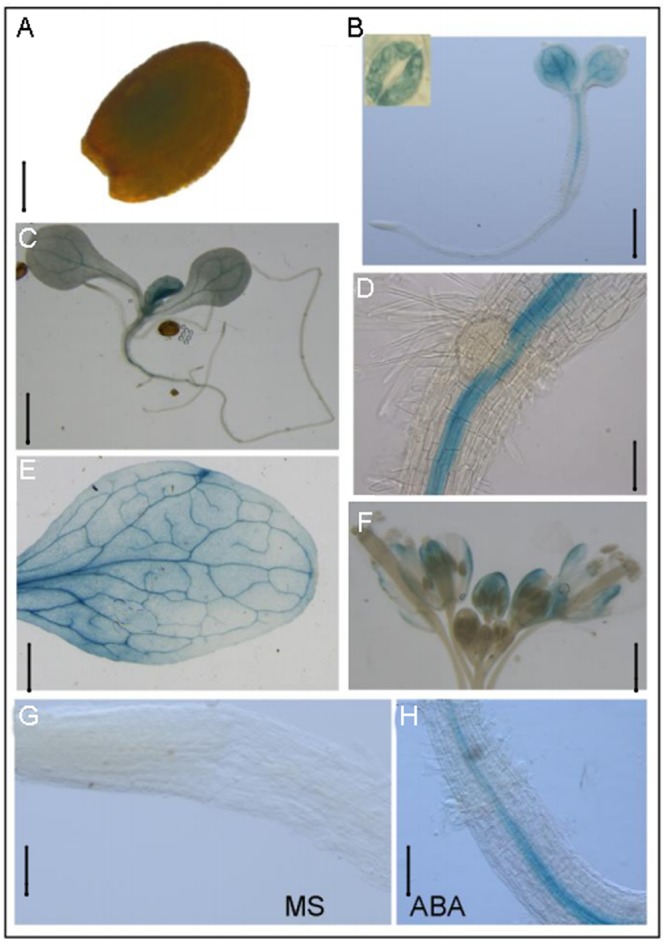
***RBM25* is ubiquitously expressed.** Histochemical analysis of *RBM25*. The GUS gene driven by the *RBM25* promoter was expressed in **(A)** imbibed seed (Bar = 0.16 mm), **(B)** 4-day-old seedling (Bar = 0.5 mm), **(C)** 8-day-old seedling (Bar = 0.5 mm), **(D)** root (Bar = 0.5 mm), **(E)** mature leaf (Bar = 2.5 mm), **(F)** inflorescence (Bar = 1 mm), **(G)** root tip in normal condition (Bar = 0.3 mm), and **(H)** root tip with ABA treatment (Bar = 0.3 mm).

Our previous results have shown that *RBM25* was expressed in all organs during plant growth and development using qRT-PCR (Wang et al., 2015). To further determine the tissue and cell-specific pattern of *RBM25* expression, we analyzed *P_RBM25_::GUS* transgenic plants. Histochemical staining of the *P_RBM25_::GUS* plants showed that *RBM25* was expressed at all developmental stages from seed germination to reproductive growth, which is consistent with the previous result (Wang et al., 2015). High levels of *RBM25* transcripts were observed in imbibed seed (**Figure 1A**), young seedling (**Figures 1B–D**), mature leaf (**Figure 1E**), and inflorescence (**Figure 1F**). In the germinating seedlings, *RBM25* was mainly expressed in cotyledons and vascular of hypocotyl; in young seedlings, *RBM25* was preferentially expressed in leaf vascular and in the vascular tissues at the shoot-root junction site (**Figures 1B–E**).

Previously, our qPCR results showed that *RBM25* is responsive to ABA in young seedlings (Wang et al., 2015). To further check the expression pattern of *RBM25*, we treated 8-day-old seedlings with or without 50 μM ABA treatment for 3 h for GUS staining. *GUS* staining showed that *RBM25* expression was substantially increased in vascular of root tip (**Figures 1G,H**). The result confirms that *RBM25* is induced by ABA and reveals that RBM25 mainly functions at the vascular tissues of plants. Together, these results suggest that *RBM25* plays crucial roles in ABA-mediated responses.

### *RBM25* Mutation Improves Plant Tolerance to Salt, Osmotic and Drought Stresses

**FIGURE 2:**
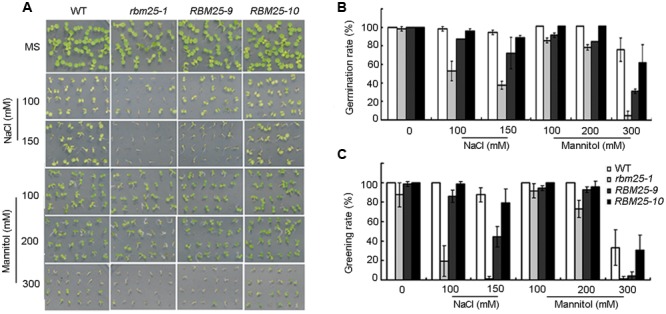
***RBM25* improves plant tolerance to salt and osmotic stress. (A)** Phenotypic analyses of wild type (WT), *rbm25-1* and the complementation lines *RBM25-9* and *RBM25-10* treated with different concentration of NaCl or mannitol. The images were taken after 4 days after germination. **(B)** The germination rates and **(C)** greening rates of WT, *rbm25-1* and the complementation lines *RBM25-9* and *RBM25-10* plants. The data were given as means plus the standard deviation of three independent replicates.

Previously we have shown that RBM25 mediates plant response to ABA and the loss-of-function mutant *rbm25* showed enhanced sensitivity to ABA during seed germination and cotyledon greening (Wang et al., 2015). Since *RBM25* was ubiquitously expressed in plants and was induced by ABA, we questioned whether RBM25 mediates plant response to abiotic stress, such as drought, osmotic and high salinity. To this end, we first germinated the seeds of *rbm25-1*, the complimented lines *RBM25-9/RBM25-10* and the wild type (WT) Col-0 on the MS medium with or without 100 or 150 mM NaCl, and evaluated the plant response to NaCl during germination and post-germination stages. In the absence of NaCl, the germination and cotyledon greening rates of the *rbm25-1* mutant were comparable to the complementary lines and the WT (**Figures 2A–C**). By contrast, in the presence of NaCl, the germination and greening rates of *rbm25-1* was significantly lower than that of the WT; while the complemented lines exhibited similar germination and greening rates to the WT (**Figures 2A–C**). We then germinated the seeds of these genotypes on MS medium supplemented with different concentration of mannitol to analyze the plant response to osmotic stress. As shown in **Figure 2**, *rbm25-1* mutant also displayed increased sensitivity to mannitol compared with the WT, whereas the complementary lines showed similar phenotypes during seed germination and cotyledon greening (**Figures 2A–C**). The results suggest *RBM25* is involved in plant responses to salt and osmotic stress during early development.

**FIGURE 3:**
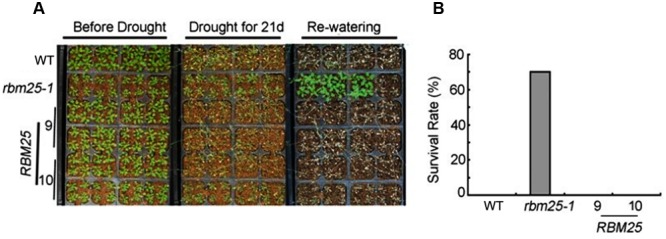
**RBM25 functions in drought tolerance. (A)** Drought tolerance assay of the 2-week-old seedlings of WT Col-0, *rbm25-1*, and the complementation lines *RBM25-9* and *RBM25-10*. **(B)** Survival rate of the Col-0, *rbm25-1, RBM25-9*, and *RBM25-10* under water stress. Three experiments were repeated with similar results, and one representative result was shown.

Next, we analyzed the phenotype of *rbm25-1* and the complementary lines under drought conditions. We transplanted 8 days-old seedlings of *rbm25-1, RBM25-9, RBM25-10* and the WT to the soil. One week after transplanting, the seedlings were subjected to drought stress by withholding water for 21 days. As shown in the **Figure 3**, all the plants of the WT and the complementary lines were severely wilted, while the *rbm25-1* plants were more vigorous. Intriguingly, when the plants were rewatered, *rbm25-1* plants exhibited a high survival rate (70%), in sharp contrast, none of the WT and the complementary lines survived (**Figures 3A,B**). This result suggests that RBM25 plays an important role in plant drought tolerance.

### RBM25 Modulates ABA-Mediated Stomatal Closure

**FIGURE 4:**
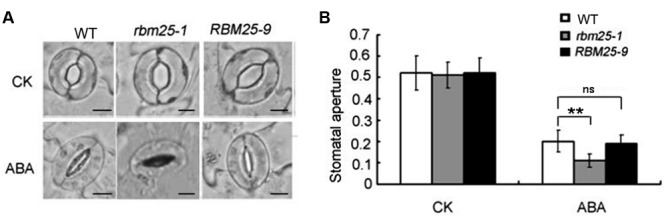
**RBM25 modulates ABA-mediated stomatal closure. (A)** Stomatal apertures of the Col-0, *rbm25-1* and *RBM25-9*. Leaves of the Col-0, *rbm25-1* and *RBM25-9* were treated with 10 μM ABA for 3 h. CK represents leaves without ABA treatment. Bar = 0.1 mM. **(B)** Stomatal apertures of the Col-0, *rbm25-1* and *RBM25-9* corresponding to **(A)**. Values are mean ratios of width to length ± standard deviations of three independent experiments (*n* = 30). The Student’s *t*-test was performed and the statically significant treatments were marked with (^∗∗^*P* < 0.01). ns, no significant (*P* > 0.05).

Stomata closure controls water evaporation and drought tolerance in plants in response to water stress. The fact that *RBM25* was highly expressed in stomata and mature leaves (**Figures 1B,E**) made us curious about whether RBM25 regulates plant drought tolerance through stomata regulation. To test the possibility, we first examined the stomatal density of the mutant. The result showed that there was no significant difference between Col-0, *rbm25-1* and the complementary line *RBM25-9* (Supplementary Figure S1). We then examined the stomatal apertures of WT, *rbm25-1* and the complementary line *RBM25-9* plants. In the absence of ABA, the stomata opening of *rbm25-1* mutant was comparable to that of WT and the complementary line *RBM25-9* plants when they were treated with KCl (**Figures 4A,B**). However, when treated with 10 μM ABA, the guard cells of *rbm25-1* mutant showed substantially enhanced sensitivity to ABA and the stomatal aperture was smaller than that of the WT (**Figures 4A,B**); while the stomatal aperture of the *RBM25-9* plants was similar to that of the WT (**Figures 4A,B**). Taken together, these results suggest that RBM25 regulates drought tolerance by modulating ABA-mediated stomatal closure.

### RBM25 Affects the Ratio of *HAB1.2/HAB1.1* under Drought Treatment

**FIGURE 5:**
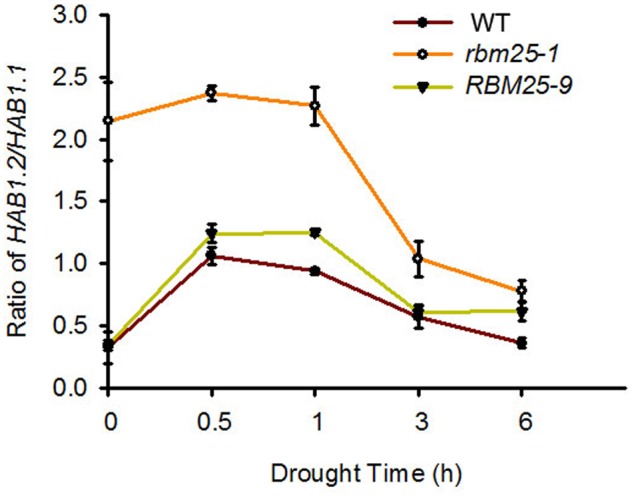
**The ratio of *HAB1.2/HAB1.1* in Col-0, *rbm25-1*, and *RBM25-9*.** RNA was extracted from 3-week-old seedlings exposed to air for indicated time, and qRT-PCR was performed. Three independent experiments were performed with similar results, each with three replicates. Values are mean ± SE.

As *rbm25-1* plants were sensitive to ABA and tolerant to drought, and our previous report found that RBM25 affects the ratio of *HAB1.2/HAB1.1* to modulate ABA signaling pathway (Wang et al., 2015), we hypothesized that RBM25 may regulate drought tolerance through the same mechanism. To test this hypothesis, we analyzed the expression of *HAB1.1* and *HAB1.2* and the ratio of *HAB1.2/HAB1.1* in the WT, *rbm25-1* and the complementary line *RBM25-9* plants. Three-week-old seedlings were taken out from the soil and exposed to air for indicated time to simulate drought. Total RNA was then extracted and qPCR was used to check the expression levels of *HAB1.1* and *HAB1.2*. As shown in the **Figure 5**, under normal conditions, the ratio of *HAB1.2/HAB1.1* in *rbm25-1* was up to about 2.2, whereas the ratios of *HAB1.2/HAB1.1* in both Col-0 and complementary line *RBM25-9* were lower than 0.5. Under drought treatment, the ratios of *HAB1.2/HAB1.1* increased at 0.5 h and then decreased as time goes on in the WT, *rbm25-1* and the complementary line *RBM25-9* plants. However, the ratio of *HAB1.2/HAB1.1* in *rbm25-1* was significantly higher than that in Col-0 and *RBM25-9* at all the time points (**Figure 5**). This result indicates that under drought stress RBM25 affects the ratio of *HAB1.2/HAB1.1* that may modulate plant response to drought stress.

### *RBM25* is Responsive to Multiple Abiotic Stresses

**FIGURE 6:**
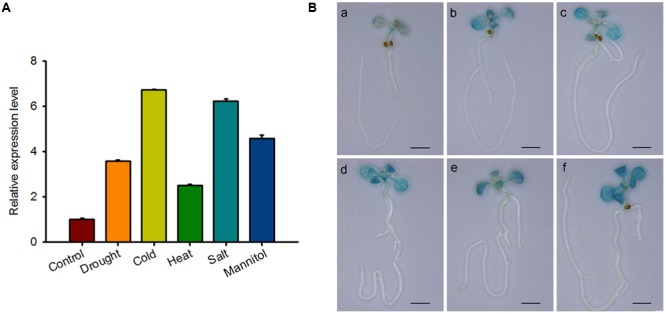
***RBM25* is responsive to multiple abiotic stresses. (A)** Ten-day-old seedlings were treated with drought (exposed to air for 5 min), cold (4°C for 6 h), heat (37°C for 6 h), salt (150 mM NaCl for 6 h) and osmotic stress (300 mM Mannitol for 6 h). RNA was extracted and qRT-PCR was performed. Three independent experiments were performed and each with three replicates. Values are mean ± SE. **(B)** Histochemical analysis of 10-day-old *P_RBM25_::GUS* seedlings treated with different stress as described in **(A)**. Bar = 2 mm.

The RNA-seq results of *RBM25* mutant reveal that in addition to *HAB1*, RBM25 also affects expression of other genes (Zhan et al., 2015), indicating that RBM25 may also participate in other biological processes in *Arabidopsis*. As plants are sessile organisms, we speculated that RBM25 may play crucial roles in *Arabidopsis* adaptation to constantly changing environmental conditions. To test the possibility, we first analyzed the expression patterns of *RBM25* in *Arabidopsis* treated with various abiotic stresses by collecting HiSeq data from the eFP website^4^. As shown in Supplementary Figure S2, *RBM25* was induced by almost all of the abiotic stresses including osmotic, drought, genotoxic, oxidative, wounding and salt stresses, although there were some variations in its expression patterns (Supplementary Figure S2). To further test the result, we first detected the expression of *RBM25* in response to different abiotic stresses. The result showed that *RBM25* expression was up-regulated under drought, cold, heat, salt and osmotic stresses (**Figure 6A**). We then performed GUS staining for the *P_RBM25_::GUS* seedlings treated with drought, cold, heat, salt, and osmotic stresses. The result confirmed that *RBM25* is induced by multiple abiotic stresses (**Figure 6B**). Interestingly, the HiSeq data indicates that *RBM25* is induced significantly in root, while our GUS staining result showed that the induction of *RBM25* expression preferentially occurred in shoots of the stressed plants (**Figure 6B**). This difference is likely due to the differences in seedling ages and stress treatments. Taken together, the results indicate that *RBM25* may play a key role in plant responses to abiotic stresses.

### The Promoter of *Arabidopsis RBM25* Contains Manifold *cis* Regulatory Elements

**FIGURE 7:**
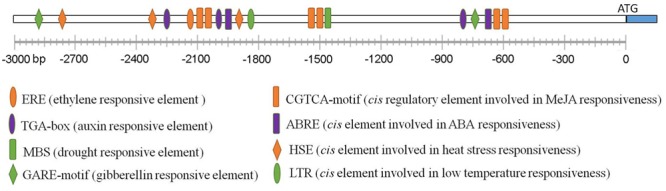
**The promoter analysis of *Arabidopsis RBM25*.** The 3-Kb DNA fragment upstream of the ATG staring code of the *Arabidopsis RBM25* gene were analyzed using PlantCARE (http://bioinformatics.psb.ugent.be/webtools/plantcare/html/).

To further understand how *RBM25* expression is regulated, we performed a promoter analysis to identify regulatory *cis*-elements using PlantCARE^3^. As shown in **Figure 7**, manifold *cis* regulat- ory elements were identified in a 3 kb DNA fragment upstream of the ATG start code of the *RBM25* gene. As expected, there are two ABRE *cis* elements (CACGTG) which is involved in ABA response and one *cis* element (MBS: TAACTG) that is related to plant response to drought in the *RBM25* promoter. It also contains several *cis* elements that are related to plant response to heat (HSE: AAAAAATTTC) and low temperature (LTR: CCGAAA). Unexpectedly, the *RBM25* promoter contains *cis* elements involved in hormone signaling which functions in plant growth regulation and response to stresses, including auxin, gibberellin acid (GA), ethylene, and methyl Jasmonate acid (MeJA). Notably, the *RBM25* promoter contains six *cis* regulatory elements involved in JA responsiveness. These observations implicate that *RBM25* is an important player in plant responses to drought and unfavorable temperatures and it may modulate plant plastic development through hormonal regulation under stresses.

### Phylogenetic and Protein Structure Analyses of *Arabidopsis RBM25* and its Homologous Genes in Plants

Previously we have shown that RBM25 is highly conserved to human RBM25 (Wang et al., 2015). To investigate whether *RBM25* plays a conserved role in plants, we used *Arabidopsis RBM25* as a query to against the genome and PGDD website to analyze the data collected from the Phytozome website^1^ and we found 14 homologous genes of *Arabidopsis* RBM25 including those from major crops, e.g., soybean, rice, maize, edible rape, and so on. Apparently, both monocots and dicots contain homologs of *Arabidopsis* RBM25, indicating that *RBM25* is a conserved gene in plants.

**FIGURE 8:**
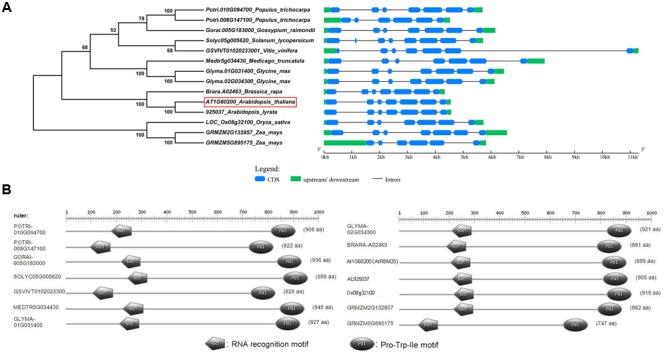
**Phylogenetic relationships and conserved protein domains analyses of *Arabidopsis* RBM25 homologous proteins. (A)** The phylogenetic tree (left panel) was constructed using MEGA6.0, and the gene structures were created using the GSDS website. **(B)** The conserved domains of the homologous proteins of *Arabidopsis* RBM25 in different species were analyzed using the PROSITE website (http://prosite.expasy.org/prosite.html).

To assess the phylogenetic relationships among the homologous genes, we constructed a phylogenetic tree using the neighbor-joining method in MEGA6.0. Among the homologs, *925031* from *Arabidopsis lyrata* and *A02463* from *Brassica rapa* are closest to *Arabidopsis RBM25* in the phylogenetic tree. Interestingly, we found two *Arabidopsis RBM25* homolog genes in the soybean genome (*Glyma.01G031400 and Glyma.02G034300*) which show a close relationship to *Arabidopsis RBM25*. To gain better understanding of these *RBM25* homolog genes, we downloaded the mRNA and genomic DNA sequences from the Phytozome database and analyzed the structures of these *RBM25* and its homologous genes using GSDS 2.0^2^. As shown in **Figure 8A**, the structures of the homologous genes are highly conserved and have a similar exon–intron pattern. All the *RMB25* homologous genes contain seven exons and six introns, although there are variations in the lengths of exons or introns (**Figure 8A**).

*Arabidopsis* RBM25 is a RNA binding protein and functions as a splicing factor (Wang et al., 2015; Zhan et al., 2015). To obtain detailed information about these homologous proteins, deduced amino acid sequences of these RBM25 homolog proteins collected from the Phytozome database were aligned, and the protein structures were analyzed using PROSITE^5^. The result showed that the length of these proteins ranges from 747 aa to 958 aa and the structures are highly conserved with a RRM domain (an RRM) at its N terminal and a PWI domain (Pro-Trp-Ile) at its C terminal (**Figure 8B**). Both RRM and PWI motifs are capable of binding to DNAs and/or RNAs. The high degree of conservation among these homologous RBM25 proteins highlights that these RBM25 proteins may share highly conserved functions, including the RNA binding activity, AS of pre-mRNA and so on.

### The Promoters of Soybean *RBM25* Genes Contain both Common and Unique *cis* Regulatory Elements

**FIGURE 9:**
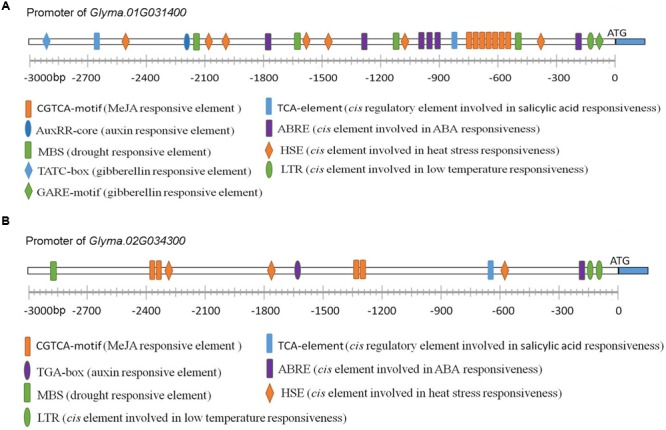
**Promoter analysis of two *RBM25* genes in soybean.** The promoters of the genes (3,000 bp) were analyzed using PlantCARE (http://bioinformatics.psb.ugent.be/webtools/plantcare/html/) to search for *cis*-elements. **(A)** The promoter of *Glyma.01G031400*. **(B)** The promoter of *Glyma.02G034300*.

The bioinformatics analysis data indicate that these *RBM25* genes and the corresponding proteins are highly conserved in plants. The next question is whether these genes or proteins have their unique characteristics that can differentiate one from another. Then we attempted to see whether there are differences between these genes at transcriptional levels. To this end, we compared the promoters of soybean *RBM25* homologs with *Arabidopsis RBM25* promoter as an example. We found that both promoters of soybean *RBM25* homologous genes contain *cis* regulatory elements related to auxin, GA, MeJA, ABA and the motifs involved in plant responses to drought and temperatures (**Figure 9**), which is similar to that of the promoter of *Arabidopsis RBM25* (**Figure 7**). The observation suggests that these *RBM25* genes are regulated by a common regulatory mechanism at the transcriptional level. However, we also found the differences between soybean and *Arabidopsis RBM25* promoters. The major difference between *Arabidopsis* and soybean *RBM25* promoters is that the former contains ethylene responsive *cis* element, while the latter does not have; however, the latter contains the *cis* element involved in SA responsiveness, whereas the former does not have. Notably, both soybean *RBM25* promoters contain the *cis* regulatory element related to SA and lack the elements involved in ethylene. These results suggest that in addition to the conserved functions, the RBM25 homolog genes in different species may have their own unique features that are related to the species. The fact that two soybean *RBM25* promoters are different in the numbers and the positions of each *cis* element indicates that the duplicates of the *RBM25* homolog genes may function or may be regulated differently.

## Discussion

Pre-mRNA AS is an important process that regulates gene expression. It’s reported that about 61% intron-containing genes of *Arabidopsis* are subjected to AS under normal conditions (Marquez et al., 2012), suggesting the importance of AS in regulating gene expression during plant growth and development. When plants are exposed to abiotic stresses, the AS events of pre-mRNAs substantially increase (Filichkin and Mockler, 2012; Staiger and Brown, 2013). Thus, it has been proposed that pre-mRNA AS is an important post-transcriptional regulation of genes that meditate plant adaptation to changing growth conditions. RBM (RNA-binding motif) proteins are important regulators in the process of AS, and many of which are involved in the response of plant to growth and environmental stimuli (Reddy et al., 2013). Previously we and other labs have shown that RBM25 in *Arabidopsis* plays a crucial role in ABA signaling at the early developmental stage (Wang et al., 2015; Zhan et al., 2015). Here, we show that RBM25 is a positive regulator of plant tolerance to abiotic stresses, such as drought and salt stress. The *RBM25* genes and proteins are highly conserved in the higher plants and their expression are possibly responsive to various hormones and abiotic stresses. Our results demonstrate that RBM25 play a conserved function in plant responses to abiotic stresses.

Previously, we have shown that *RBM25* is responsive to ABA and modulates plant response to ABA during seed germination and post-germinative growth in *Arabidopsis* (Wang et al., 2015). Apparently, *RBM25* is not only expressed in the early development of *Arabidopsis*. Our histochemical analysis results clearly show that *RBM25* was expressed in various tissues and organs in the whole life cycle of *Arabidopsis* and was responsive to ABA (**Figure 1**), which is consistent with our qRT-PCR data (Wang et al., 2015). This expression patterns of *RBM25* implicit its roles in plant responses to abiotic stresses during late developmental stages. In this study, we provide several lines of evidences to support the notion that *RBM25* is a key regulator of plant responses to abiotic stresses. Firstly, RBM25 is required for plant responses to osmotic stresses during early development of *Arabidopsis*, because the *rbm25* mutant plants were more sensitive to salt and mannitol during seed germination and post-germinative growth (**Figure 2**). Secondly, RBM25 is an important negative regulator of plant drought tolerance because loss of function in RBM25 results in greatly enhanced drought tolerance of the plants (**Figures 3, 4**). Thirdly, promoter analysis identifies several *cis*-elements related to abiotic stresses (dehydration, low temperature, etc.) in *RBM25* promoter, including ABRE, MBS, HSE, LTR (**Figure 7**), suggesting that expression of *RBM25* is responsive to the abiotic stresses. Finally, the expression data from the public website, our qRT-PCT and GUS staining assay results show that *RBM25* was induced by abiotic stresses, including osmotic, drought, genotoxic, oxidative, wounding, and salt stresses (**Figure 6** and Supplementary Figure S2). These results demonstrate that RBM25 play an important role in the response to abiotic stresses in *Arabidopsis*.

Previously, we have shown that RBM25 modulates seed germination and post-germinative growth in response to ABA through regulating AS of *HAB1* pre-mRNA that produces two isoforms of HAB1, HAB1.1 and HAB1.2 with opposite functions in SnRK2.6 (SNF1-RELATED PROTEIN KINASE 2.6) regulation (Wang et al., 2015). HAB1.1 can interact with SnRK2.6 and inhibits the kinase activity of SnRK2.6 and by dephosphorylation of the protein, thereafter switching off the ABA signaling; while HAB1.2 interacts with SnRK2.6 but keeps SnRK2.6 in an active state and the ABA signaling on (Wang et al., 2015). Here, we show that RBM25-mediated drought tolerance is partially dependent on the alterative splicing of pre-mRNA of *HAB1*. The ratio of *HAB1.2/HAB1.1* in the *rbm25* mutant plants was much higher than that in the WT and complementary plants in response to drought treatment, and accordingly, the mutant plants exhibited dramatically smaller stomatal aperture and increased tolerance to drought (**Figures 3, 4**). As RBM25 affects AS of many genes based on the RNA-seq data (Zhan et al., 2015), we do not exclude the possibility that RBM25 also modulates plant drought tolerance via regulating AS of other genes that are responsible for the drought tolerance of plants. So far, we still do not know how RBM25 mediates plant responses to other abiotic stresses, such as low temperature. It is likely that RBM is in charge of AS of the genes involved in different abiotic stresses. Further identification and characterization of the target genes whose pre-mRNA AS are regulated by RBM25 in response to various stress conditions will help us to understand RBM25-mediated plant tolerance to various abiotic stresses in *Arabidopsis*.

It is worthy to note that RBM25 is highly conserved proteins in plants. These RBM25 proteins in different plant species share similar gene and protein structures. All the RBM25 proteins contain a RRM in the N-terminal ends and a PWI (Pro-Leu-Ile) motif in the C-terminal ends (**Figure 8**) which can bind to RNA or DNA. The high level of the protein identity of these RBM25 suggests a conserved role for these proteins in plant responses to various abiotic stresses. Comparison between *Arabidopsis RBM25* and two soybean *RBM25* homolog promoters favors idea that *RBM25s* are responsive to various abiotic stresses because both *Arabidopsis* and soybean *RBM25* genes contain many *cis*-elements, including ABRE, MBS, HSE, and LTR in their promoters. Besides the stress-related *cis*-elements, there are also some elements involved in hormonal responses in plants, including CGTCA-motif responsive to methyl jasmonate, AuxRR-core to auxin, TATC-box and GARE-motif to gibberellin and TCA-element to salicylic acid. Multiple *cis* regulatory elements related to abiotic stress and hormonal responses in the promoters of *RBM25* genes implicate that these RBM25 may function as a molecular node that integrates hormonal and abiotic signals to orchestrate plant adaptation to the changing environment. Interestingly, we also found some different *cis* elements (TCA-element to salicylic acid and ERE-element to ethylene), which are unique to *Arabidopsis* or soybean *RBM25* genes (**Figures 7, 9**), suggesting that functional diversity of *RBM25* also exists in different plant species. In summary, RBM25-mediated AS of pre-mRNAs plays a key role in plant response to abiotic stresses. Our results provide an overview of the main characteristics of these *RBM25* genes and their potential functions in different plant species. Most importantly, these findings will facilitate our understanding of how plants tolerate abiotic stresses and provide novel insight into genetic improvement of plant stress tolerance.

## Author Contributions

XL and ZW conceived the project and designed the experiments. ZW, CC, and BY performed the experiments, analyzed the data; ZW, CC, and XL prepared the manuscript.

## Conflict of Interest Statement

The authors declare that the research was conducted in the absence of any commercial or financial relationships that could be construed as a potential conflict of interest.

## Footnotes

1 https://phytozome.jgi.doe.gov/pz/portal.html

2 http://gsds2.cbi.pku.edu.cn

3 http://bioinformatics.psb.ugent.be/webtools/plantcare/html/

4 http://bbc.botany.utoronto.ca/efp/cgi-bin/efpWeb.cgi

5 http://prosite.expasy.org/prosite.html
