# Supplementary material for: RBM25 Mediates Abiotic Responses in Plants
Source: Front Plant Sci. 2017 Mar 10;8:292. doi: 10.3389/fpls.2017.00292 (PMC5344909; doi:10.3389/fpls.2017.00292)
Supplement: Supplementary file 1 [file Data_Sheet_1.pdf]

Supplementary Material

RBM25 mediates abiotic responses in plants

Chunhong Cheng[1,2†], Zhijuan Wang[3†], Bingjian Yuan[1,2], Xia Li[3*]

[†]These authors equally contributed to this work.
[*]Corresponding author: xli@mail.hzau.ac.cn

Supplementary Information
Supplementary Figure S1 Comparison assay of stomatal density of WT, *rbm25-1* and *RBM25-9*.
Supplementary Figure S2 Heat map presentation for the expression level of *Arabidopsis RBM25* induced by different abiotic stresses.
Supplementary Table S1 The sequences of the primers used in this study.

Methods

Stomatal Density Measurement

For stomatal density measurement, fully expanded 3-week-old leaves from Col-0, *rbm25-1* and *RBM25-9* were used. The number of stomata was determined in a 0.138 mm^2 area using an abaxial epidermal peel taken from the middle of each leaf. The epidermis was printed using Super Glue (Ontario, CA) and the imprint on the glass slide was observed with a confocal microscope (Leica TCS-SP8; Leica Microsystems).

Expression Data Collection and Heatmap Construction

The heat map was generated using MeV software, v. 4.9. The raw data was downloaded from the website http:// bbc.botany.utoronto.ca/efp/cgi-bin/efpWeb.cgi .

Statistical Analysis

All data were analyzed using SigmaPlot 10.0 (Systat Software, Inc., Chicago, IL). The averages and standard deviations of all results were calculated, and Student's t-tests were performed to generate P values. Same letters indicate no significant difference in the relative gene expression ($P > 0.05$)

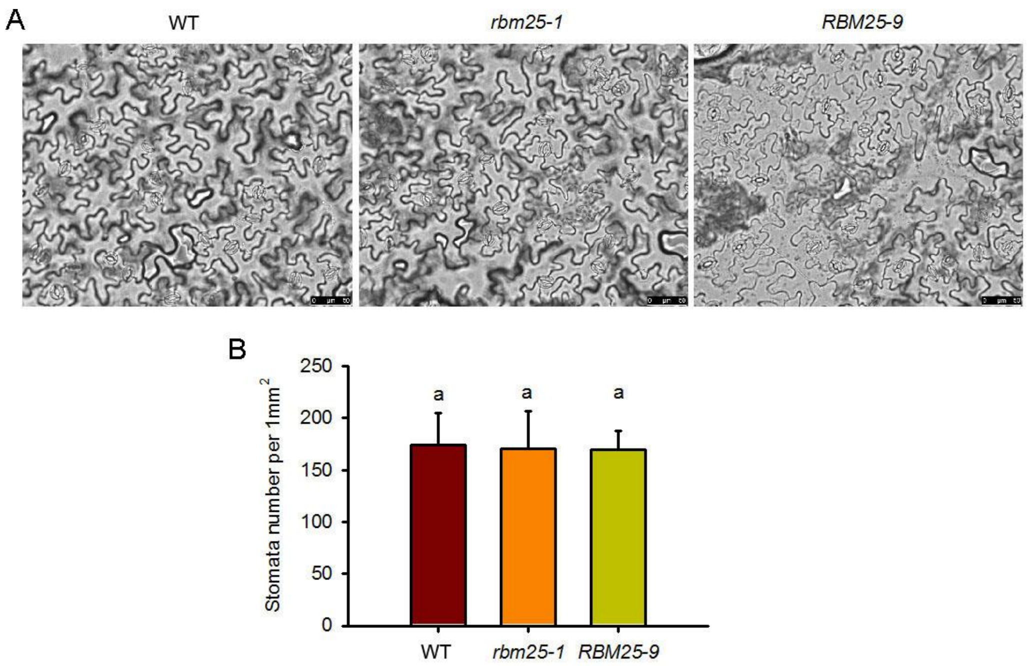

Supplementary Figure S1 Comparison assay of stomatal density of WT, *rbm25-1* and *RBM25-9*. (**A**) An abaxial epidermal peel in the middle of the leaves from WT, *rbm25-1* and *RBM25-9* plants was printed and photographed to show the stomatal density. Scale bar = 50 μm. (**B**) The number of stomata per 0.138mm^2 in the abaxial epidermis of fully expanded rosette leaves (3-week-old plants, 16 h/8 h photoperiod). All values are the means ± standard deviations of three independent experiments. "a" indicates no significant difference (Student-Newman-Keuls test, $P > 0.05$).

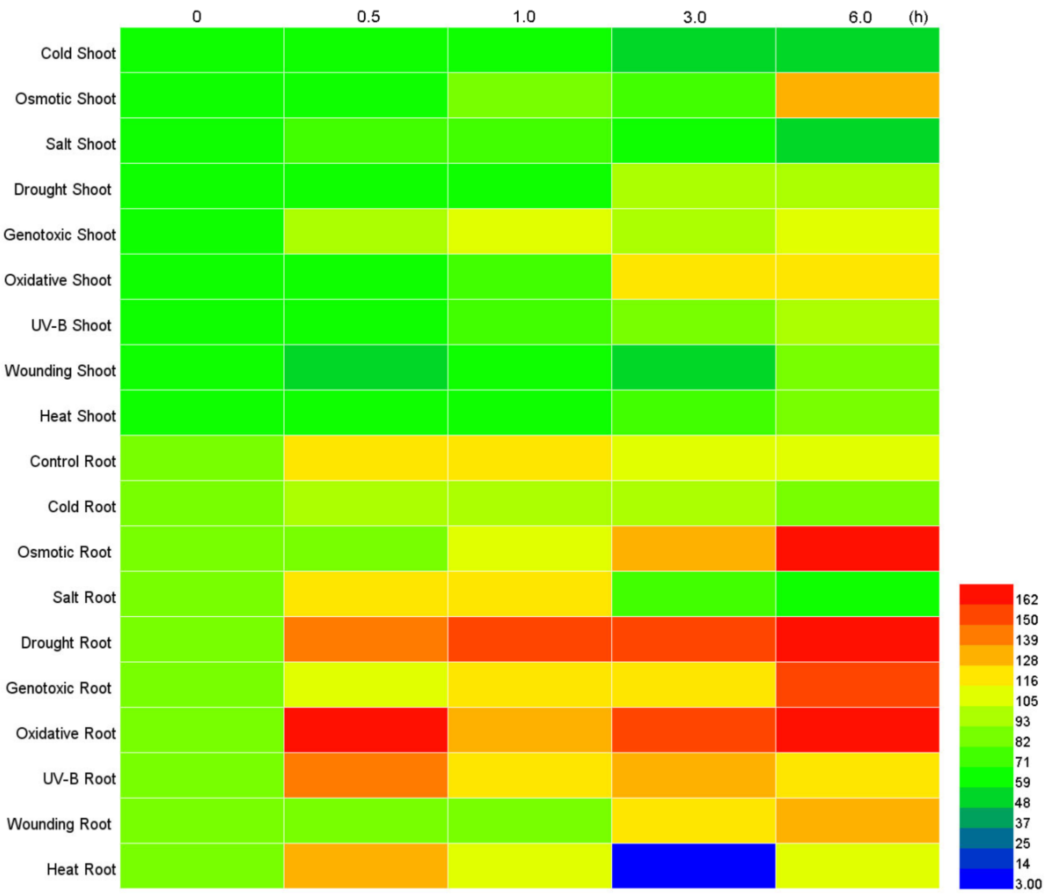

Supplementary Figure S2 The heat map was generated using MeV software, v. 4.9. The value at the right panel indicated the expression level. The raw data was downloaded from the website http://bbc.botany.utoronto.ca/efp/cgi-bin/efpWeb.cgi.

Supplementary Table S1 The sequences of the primers used in this study.

Application	Name	Sequence (5'-3')
Promoter analysis	Pro-RBM25-F	GGATCCAGAGTCTCTTCCTCACAAACAAACA
	Pro-RBM25-R	GAATTCGGGAGTTTTGGTTACAAGCTTTGGC
qPCR	HAB1.1-F	CTAGGTCCATCGGTGACAGATATC
	HAB1.1-R	TCATTACATCCCAAAGACCGTC
	RBM25-F	CTCCGGTATCCTTCTCCATATCC
	RBM25-R	TGAACAGCACCGATTGTACCA
	HAB1.2-F	CTTTCCTCCTCTGGAACTATGCAG
	HAB1.2-R	TCATTACATCCCAAAGACCGTC
RBM25 genome-GFP complementation rescue	RBM25-F	GGACTAGT ATGGCCGACGAATCTTCTTC
	RBM25-R	GAAGATCT GGCTTTGGATTTTACCG
rbm25-1 homozygous identification	*rbm25-1*-LP	ATTGAAAAGCACCAAGTGCAC
	rbm25-1-RP	AAATCGGAGGAGATTAGCGAC
	LBb1.3	ATTTTGCCGATTTCGGAAC
